# Maternal serum C-reactive protein concentration and intra-amniotic inflammation in women with preterm prelabor rupture of membranes

**DOI:** 10.1371/journal.pone.0182731

**Published:** 2017-08-16

**Authors:** Ivana Musilova, Marian Kacerovsky, Martin Stepan, Tomas Bestvina, Lenka Pliskova, Barbora Zednikova, Bo Jacobsson

**Affiliations:** 1 Department of Obstetrics and Gynecology, Charles University Faculty of Medicine in Hradec Kralove, University Hospital Hradec Kralove, Hradec Kralove, Czech Republic; 2 Biomedical Research Center, University Hospital Hradec Kralove, Hradec Kralove, Czech Republic; 3 Institute of Clinical Biochemistry and Diagnostics, University Hospital Hradec Kralove, Hradec Kralove, Czech Republic; 4 Department of Clinical Immunology and Allergy, University Hospital Hradec Kralove, Hradec Kralove, Czech Republic; 5 Department of Obstetrics and Gynecology, Sahlgrenska Academy, Gothenburg University, Gothenburg, Sweden; 6 Domain of Health Data and Digitalization, Norwegian Institute of Public Health, Oslo, Norway; Morehouse School of Medicine, UNITED STATES

## Abstract

**Objective:**

To evaluate maternal serum C-reactive protein (CRP) concentrations in pregnancies complicated by preterm prelabor rupture of membranes (PPROM) in relation to the presence of microbial invasion of the amniotic cavity (MIAC) and/or intra-amniotic inflammation (IAI).

**Methods:**

Two hundred and eighty-seven women with singleton pregnancies complicated by PPROM between 2014 and 2016 were included in this study. Maternal blood and amniotic fluid samples were collected at the time of admission. Maternal serum CRP concentration was measured using a high-sensitivity immunoturbidimetric assay. Interleukin-6 (IL-6) concentration was measured using a point-of-care test. MIAC was diagnosed based on a positive polymerase chain reaction result for *Ureaplasma* species, *Mycoplasma hominis*, and/or *Chlamydia trachomatis* and for the 16S rRNA gene. IAI was characterized by an amniotic fluid IL-6 concentration of ≥ 745 pg/mL.

**Result:**

Women with MIAC and IAI had higher maternal serum CRP concentrations than did women without (with MIAC: median 6.9 mg/L vs. without MIAC: median 4.9 mg/L; *p* = 0.02; with IAI: median 8.6 mg/L vs. without IAI: median 4.7 mg/L; *p* < 0.0001). When women were split into four subgroups based on the presence of MIAC and/or IAI, women with the presence of both MIAC and IAI had higher maternal serum CRP than did women with IAI alone, with MIAC alone, and women without MIAC and IAI (both MIAC and IAI: median: 13.1 mg/L; IAI alone: 6.0 mg/L; MIAC alone: 3.9 mg/L; and without MIAC and IAI: median 4.8 mg/L; *p* < 0.0001). The maternal serum CRP cutoff value of 17.5 mg/L was the best level to identify the presence of both MIAC and IAI, with sensitivity of 47%, specificity of 96%, positive predictive value of 42%, negative predictive value of 96%, and the positive likelihood ratio of 10.9.

**Conclusion:**

The presence of both MIAC and IAI was associated with the highest maternal serum CRP concentrations. Maternal serum CRP concentration in women with PPROM at the time of admission can rule out the presence of the combined condition of both MIAC and IAI, therefore, it may serve as a non-invasive screening tool to distinguish between women with PPROM who are at high or at low risk for the presence of both MIAC and IAI.

## Introduction

Preterm prelabor rupture of membranes (PPROM) is characterized by the rupture of fetal membranes with leakage of amniotic fluid before spontaneous onset of regular uterine contractions prior to 37 weeks of gestation. PPROM complicates approximately 2–4% of all pregnancies [1, 2]. In spite of growing knowledge about the etiologies of PPROM, this pregnancy complications remains a challenge for obstetricians, owing to the limited possibility of predicting and preventing the condition. Thus, PPROM still represents a serious problem in contemporary obstetrics.

Pregnancies with PPROM are often complicated by adverse intra-amniotic conditions such as microbial-invasion of the amniotic cavity (MIAC) and intra-amniotic inflammation (IAI) [3–6]. MIAC and IAI are found in approximately 40–60% and 25–58% of all pregnancies with PPROM, respectively [4, 5]. The presence of these complications is associated with worse outcomes such as shorter latency and higher rates of histological chorioamnionitis or funisitis [3, 4, 7]. Several authors have suggested that gestational age at delivery but not the presence of these infection-related and inflammatory complications affect short-term neonatal outcomes [5, 8, 9]. In contrast, longer exposure to a hostile environment has been shown to be related to worse neonatal outcomes [10, 11]. To address whether MIAC and IAI really affect the fetuses, data regarding these conditions and long-term outcome are needed.

C-reactive protein (CRP) is an acute phase protein produced and released in the circulation in response to infection and tissue damage [12, 13]. CRP belongs to a family of the proteins that act as soluble pattern recognition molecules [14, 15]. These proteins are able to bind directly to microorganisms to enhance their uptake by macrophages and neutrophils [13]. In addition, CRP is able to activate the complement system [13–15].

In many countries, maternal serum CRP is still considered the gold standard for non-invasive identification of infection-related intra-amniotic complications in PPROM, despite that results from two meta-analyses revealing that maternal serum CRP was not a useful predictor of histological chorioamnionitis (HCA) [16, 17]. Our group has recently published a report on about maternal serum CRP concentrations at the time of admission in the subgroup of women with MIAC and/or HCA [18]. The presence of both MIAC and HCA has been shown to be related to the highest maternal serum CRP concentrations; however, CRP had poor the diagnostic indices to identify this subgroup of PPROM [18].

Since expectant management of PPROM less then 34 weeks of gestational age is broadly recommended, the placental results cannot be correlated with amniotic fluid results due to the long latency between sampling and delivery [19]. From this point of view, subgroups of women with PPROM divided by the presence of MIAC and/or IAI, as suggested by Romero et al.’s study, more appropriately reflect a real situation [4]. Given this subdivision of women with PPROM, there is a shortage of information regarding the intensity of maternal inflammatory response, measured by maternal serum CRP concentrations, in these specific subgroups.

Therefore, the main aim of this study was to determine maternal serum CRP concentrations in women with and without MIAC and IAI. The second aim was to characterize maternal serum CRP concentrations in four subgroups of women with PPROM subdivided on the basis of the presence of MIAC and/or IAI. The last aim of this study was to assess the association between maternal serum CRP and amniotic fluid IL-6 concentrations.

## Materials and methods

A prospective cohort study was conducted between January 2014 and December 2016. Women admitted to the Department of Obstetrics and Gynecology, University Hospital in Hradec Kralove, The Czech Republic were recruited if they had pregnancies complicated by PPROM between gestational ages 24+0 and 36+6 weeks. Only women aged at least 18 years and older with a singleton pregnancy were eligible for the study. Women with any medical complications (e.g., hypertension, preeclampsia, diabetes mellitus, and thyroid disease), fetal growth restriction, gross vaginal bleeding, signs of fetal hypoxia, and structural malformations or chromosomal abnormalities of the fetus were excluded from the study. Gestational age was established for all pregnancies based on first-trimester ultrasonography.

PPROM was defined as the leakage of amniotic fluid prior to the onset of labor and was diagnosed visually by using a sterile speculum examination to confirm the pooling of amniotic fluid in the vagina. In case of clinical doubt, PPROM was confirmed by the presence of insulin-like growth factor–binding protein (ACTIM PROM test; Medix Biochemica, Kauniainen, Finland) in the vaginal fluid.

Women with PPROM at less than 34 weeks of gestation were treated with tocolytics for 48 hours, antibiotics, and corticosteroids to accelerate lung maturation. The performance of transabdominal amniocentesis and the evaluation of amniotic fluid samples is a routine part of the clinical management of women with PPROM at our department. Amniotic fluid samples are evaluated for the presence of MIAC and/or IAI. The information about the presence of MIAC and IAI, when available, is used for the clinical management of women with PPROM. Women with both proven MIAC and IAI beyond 28 gestational weeks were actively managed (labor was induced or an elective caesarean section was performed after finalizing corticosteroid treatment within 72 hours of membrane rupture for pregnancies before 34 weeks gestational age, and within 24 hours of membrane rupture for those beyond 34 weeks). The remaining women with PPROM were managed expectantly. Women with PPROM beyond 34 weeks of gestation were treated with antibiotics alone [20].

This study’s protocol was approved by the Ethics Committee of University Hospital in Hradec Kralove, the Czech Republic (March 19, 2008; No. 200804 SO1P, and renewed in July, 2014; decision No. 201407 S14P), and written informed consent was obtained from all the participants.

Amniotic and vaginal fluid samples from this cohort of women have been used in our previously published studies [20, 21]. This cohort of women is completely different than the cohort of women used in our previous CRP report [18].

### Maternal blood amniotic fluid sampling

In all women, the maternal blood and amniotic fluid samples were collected at the time of admission (maternal blood first, followed by amniotic fluid) prior to the administration of corticosteroids, antibiotics, or tocolytics. Maternal blood sample was obtained by venipuncture of the cubital vein, and was sent to the laboratory immediately following sampling. Ultrasonography-guided transabdominal amniocentesis was carried out, and approximately 5 mL of amniotic fluid was aspirated, and a tube with uncentrifuged amniotic fluid was transported to the laboratory for DNA isolation; detection of *Ureaplasma* spp., *Mycoplasma hominis*, *Chlamydia trachomatis* using polymerase chain reaction (PCR); and 16S rRNA gene sequencing.

### Maternal serum CRP analysis

Maternal serum CRP was measured using a high-sensitivity immunoturbidimetric assay (Modular RR analyzer, Roche, Basel, Switzerland). The sensitivity of the method was 0.3 mg/L.

### Amniotic fluid IL-6 concentrations

The amniotic fluid IL-6 concentrations were assessed by the Milenia QuickLine IL-6 lateral-flow immunoassay using the Milenia POCScan Reader (Milenia Biotec, GmbH, Giessen, Germany). The measurement range was 50–10,000 pg/mL. The intra-assay and interassay coefficients of variation were 12.1% and 15.5%, respectively [22].

### Detection of *Ureaplasma* species, *M*. *hominis*, and *C*. *trachomatis*

DNA was isolated from the amniotic fluid with a QIAamp DNA Mini Kit (Qiagen, Hilden, Germany) according to the manufacturer’s instructions (using the protocol for isolating bacterial DNA from biological fluids). Real-time PCR was conducted on a Rotor-Gene 6000 instrument (Qiagen) with the commercial kit AmpliSens^®^*C*. *trachomatis/Ureaplasma/M*. *hominis*-FRT (Federal State Institution of Science, Central Research Institute of Epidemiology, Moscow, Russia) to detect the DNA of *Ureaplasma* species, *M*. *hominis*, and *C*. *trachomatis* in the same PCR tube. As a control, we included a PCR for β-actin, a housekeeping gene, to examine for the presence of PCR inhibitors [20].

### Detection of other bacteria in the amniotic fluid

Bacterial DNA was identified by PCR targeting the 16S rRNA gene with the following primers: 5′-CCAGACTCCTACGGGAGGCAG-3′ (V3 region), 5′-ACATTTCACAACACGAGCTGACGA-3′ (V6 region) [23, 24]. Each reaction contained 3 μL of target DNA, 500 nM forward and reverse primers, and Q5 High-Fidelity DNA polymerase (NEB, Ipswich, MA, USA) in a total volume of 25 μL. Amplification was performed on a 2720 Thermal Cycler (Applied Biosystems, Foster City, CA, USA). The products were visualized on an agarose gel. Positive reactions yielded amplicons of 950 bp, which were subsequently analyzed by sequencing. The PCR products from 16S rRNA were cleaned and used in sequencing PCR reactions with the above primers and the BigDye Terminator kit, v3.1 (Thermo Fisher Scientific). The bacteria were then typed using the sequences obtained in BLAST^®^ and SepsiTest^™^ BLAST.

### Diagnosis of MIAC

MIAC was diagnosed based on a positive PCR result for *Ureaplasma* species, *M*. *hominis*, and/or *C*. *trachomatis* and/or by positivity of the 16S rRNA gene.

### Diagnosis of IAI

IAI in pregnancies with PPROM was defined as bedside amniotic fluid IL-6 concentrations ≥ 745 pg/mL [25, 26]. Women were subdivided into four groups based on the presence of MIAC and/or IAI: presence of both MIAC and IAI (microbial-associated IAI), IAI alone(sterile IAI), MIAC alone (colonization), and absence of both MIAC and IAI.

### Statistical analyses

The demographic and clinical characteristics were compared by the nonparametric Mann-Whitney *U* test for continuous variables and are presented as median values (range). Categorical variables were compared using a Fisher’s exact test and are presented as numbers (%). Maternal serum CRP concentrations were compared by either the Mann-Whitney *U* test or Kruskal-Wallis test with *post hoc* Dunn’s analysis, as appropriate, and presented as median values [interquartile range (IQR)]. Spearman’s partial correlation was used to adjust the results for potential confounders [gestational age, parity, and body mass index (BMI)]. To identify an association between amniotic fluid IL-6 concentrations and maternal serum CRP concentrations, the Spearman correlations were used. Differences were considered significant at *p* < 0.05. All *p* values were obtained in two-sided tests, and all statistical analyses were performed in the GraphPad Prism 6 software for Mac OS X (GraphPad Software, San Diego, CA, USA) or the SPSS 19.0 statistical package for Mac OS X (SPSS Inc., Chicago, IL, USA).

## Results

### Demographic and clinical characteristics of the study population

**Fig 1:**
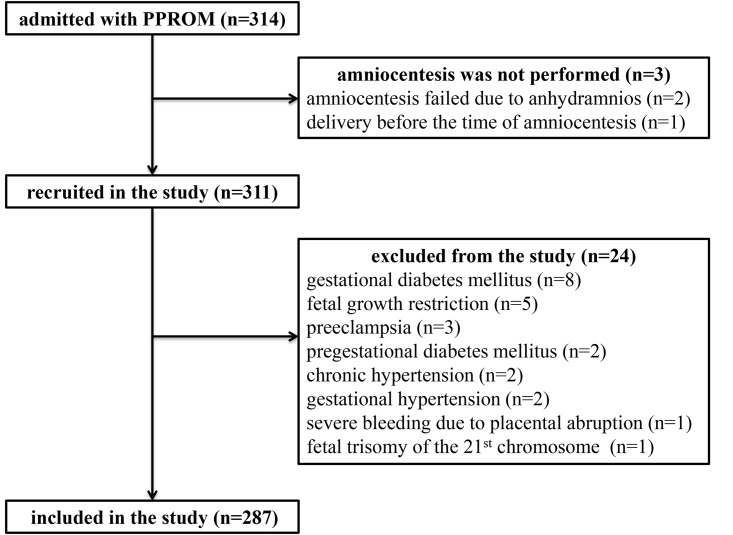
Flow diagram describing the selection of women.

In total, 314 women with singleton pregnancy complicated by PPROM were admitted with the diagnosis of PPROM during the study period. Three women were not recruited because an amniotic fluid sampling was not performed (amniocentesis failed in two women due to anhydramnios and one women delivered before the time of amniocentesis). Therefore, 313 women with PPROM were included in the study. Eight women were excluded because of gestational diabetes mellitus, five for early-onset fetal growth restriction, three because of preeclampsia, two because of pregestational diabetes mellitus, two because of chronic hypertension, two because of gestational hypertension, one because of severe bleeding due to partial placental abruption, and one because of fetal trisomy 21. A total of 287 women were included in the final analyses (Fig 1).

The presence of MIAC and IAI were found in 24% (69/287) and 20% (57/287) of women, respectively. The prevalence of the presence of both MIAC and IAI, IAI alone, and MIAC alone was 13% (36/287), 7% (21/287), and 11% (33/287), respectively. In addition, 69% (197/287) of women did not have both MIAC and IAI. The demographic and clinical characteristics of these women are shown in Table 1. The most common bacteria identified in the amniotic fluid were *Ureaplasma* spp., which was identified in 16% (46/287) of the women with PPROM. All microbial findings in amniotic fluid are presented in Table 2. All the women were self-reported Caucasians.

**Table 1:** Demographic and clinical characteristics of the women with PPROM with respect to the presence and the absence of MIAC and IAI.

Characteristic	With MIAC (n = 69)	Without MIAC (n = 218)	*p*-value^1^	With IAI (n = 57)	Without IAI (n = 230)	*p*-value^2^
Maternal age [years, median (range)]	31 (20–42)	31 (18–43)	0.22	31 (21–43)	31 (18–43)	0.59
Primiparous [number (%)]	32 (46%)	113 (52%)	0.49	29 (51%)	116 (50%)	1.00
Prepregnancy body mass index [kg/m^2^, median (range)]	22.3 (16.8–36.4)	22.3 (17.8–57.0)	0.16	23.6 (17.1–38.7)	22.4 (16.8–57.0)	0.76
Smoking [number (%)]	21 (30%)	18 (8%)	**< 0.0001**	17 (30%)	18 (8%)	**< 0.0001**
Gestational age at admission [weeks, median (range)]	33+4 (24+0–36+5)	34+1 (24+0–36+6)	0.14	31+3 (24+0–36+6)	34+3 (24+0–36+5)	**<0.0001**
Gestational age at delivery [weeks, median (range)]	33+5 (25+0–36+5)	34+3 (24+0–37+1)	0.07	31+4 (24+0–37+0)	34+4 (24+0–37+1)	**<0.0001**
Interval from PPROM to amniocentesis [hours, median (range)]	5 (1–575)	5 (1–600)	0.78	5 (1–575)	5 (1–600)	0.47
Latency from amniocentesis to delivery [hours, median (range)]	45 (4–624)	40 (4–768)	0.99	63 (4–768)	36 (4–768)	**0.04**
Amniotic fluid IL-6 concentrations [pg/mL, median (range)]	831 (50–10000)	225 (36–10000)	**<0.0001**	3588 (747–10000)	209 (36–725)	**<0.0001**
CRP levels at admission [mg/L, median (range)]	6.9 (0.4–113.0)	4.9 (0.1–59.1)	**0.02**	8.6 (0.6–113.0)	4.7 (0.1–17.0)	**<0.0001**
WBC count at admission [x10^9^ L, median (range)]	12.8 (6.8–29.1)	12.1 (6.1–26.5)	**0.04**	13.8 (9.1–29.1)	12.1 (6.1–26.5)	**<0.0001**
Administration of antibiotics [number (%)]	67 (97%)	213 (98%)	0.68	55 (96%)	225 (98%)	0.63
Administration of corticosteroids [number (%)]	51 (74%)	172 (79%)	0.41	47 (82%)	176 (77%)	0.38
Spontaneous vaginal delivery [number (%)]	52 (75%)	156 (72%)	0.64	40 (70%)	168 (73%)	0.74
Forceps delivery [number (%)]	1 (1%)	2 (1%)	0.56	0 (0%)	3 (1%)	1.00
Cesarean delivery [number (%)]	16 (23%)	60 (28%)	0.53	17 (30%)	69 (26)	0.51
Birth weight [grams, median (range)]	1980 (700–3540)	2260 (690–3670)	0.10	1580 (690–3540)	2300 (700–3670)	**<0.0001**
Apgar score <7; 5 minutes [number (%)]	3 (4%)	4 (2%)	0.36	3 (5%)	4 (2%)	0.14
Apgar score <7; 10 minutes [number (%)]	1 (1%)	3 (2%)	1.00	2 (4%)	2 (1%)	0.18

Abbreviations: PPROM: preterm prelabor rupture of membranes. MIAC: microbial invasion of the amniotic cavity. IAI: intraamniotic inflammation. CRP: C-reactive protein. WBC: white blood cells. IL: interleukin. Continuous variables were compared using a nonparametric Mann-Whitney *U* test. Categorical variables were compared using Fisher’s exact test. Statistically significant results are marked in bold. Continuous variables are presented as median (range) and categorical as number (%). *p*-value^1^: the comparison between women with and without MIAC. *p-*value^2^: the comparison between women with and without IAI

**Table 2:** The bacterial species identified in the amniotic fluid of women with PPROM.

Women with MIAC and IAI (n = 36)	Women with MIAC alone (n = 24)
*Ureaplasma* spp. 19x	*Ureaplasma* spp. 23x
*Ureaplasma* spp. + *Mycoplasma hominis* 1x	*Mycoplasma hominis* 1x
*Ureaplasma* spp. + *Sneathia sanguinegens* 1x	*Chlamydia trachomatis* 1x
*Ureaplasma* spp. + *Veilonella* spp. 1x	*Enterococcus faecalis* + *Streptococcus salivarius* 1x
*Ureaplasma* spp. + *Enterococcus faecium* 1x	*Propionibacterium acnes* 2x
*Streptococcus agalactiae* 2x	*Streptococcus pneumoniae1x*
*Fusobacterium nucleatum* 2x	*Streptococcus intermedius 1x*
*Streptococcus agalactiae + Streptococcus anginosus* 1x	*Streptococcus warneri* 1x
*Streptococcus intermedius* 1x	*Gardnerella vaginalis* 1x
*Streptococcus* spp. 1x	
*Sneathia sanguinegens* 1x	
*Peptoniphilus species* 1x	
*Haemophilus influenzae* 1x	
*Chlamydia trachomatis* 1x	
*Candida albicans* 1x	
Bacteria non-identifiable by sequencing 1x	

### Maternal serum CRP concentrations based on the presence of MIAC

Women with MIAC had higher maternal serum CRP concentrations than women without MIAC in crude analysis (with MIAC: median 6.9 mg/L, IQR: 3.0–16.1 vs. without MIAC: median 4.9 mg/L, IQR: 2.4–7.8; *p* = 0.02), as well as after adjustment for gestational age at sampling, parity, and BMI (*p* < 0.0001). A cutoff value of 7.9 mg/L was identified as optimal for the identification of MIAC with a sensitivity of 49% (34/69; 95% confidence interval [CI]: 37–62%), specificity of 71% (154/218; 95% CI: 64–77%), positive predictive value of 35% (34/98; 95% CI: 25–45%), negative predictive value of 81% (154/189; 95% CI: 75–87%), positive likelihood ratio of 1.7 (95% CI: 1.2–2.3), and negative likelihood ratio of 0.7 (95% CI: 0.6–0.9), area under the receiver operating characteristic curve of 60% (95% CI: 51–68%).

### Maternal serum CRP concentrations based on the presence of IAI

Women with IAI had higher maternal serum CRP concentrations than women without IAI in crude analysis (with IAI: median 8.6 mg/L, IQR: 4.6–26.2 vs. without IAI: median 4.7 mg/L, IQR: 2.3–7.8; *p* < 0.0001), as well as after adjustment for gestational age at sampling, parity, and BMI (*p* < 0.0001). The maternal serum CRP cutoff value of 8.6 mg/L was found to be the most effective at identifying MIAC a, with sensitivity of 51% [29/57; 95% CI 37–64%] specificity of 82% (189/230; 95% CI 77–87%), positive predictive value of 41% (29/70; 95% CI 30–54%), negative predictive value of 87% (28/189; 95% CI 82–91%), positive likelihood ratio of 2.9 (95% CI 2.0–4.2), negative likelihood ratio of 0.6 (95% CI 0.5–0.8), odds ratio of 4.8 (95% CI 2.6–8.9), and area under the receiver operating characteristic curve of 0.70 (95% CI 0.61–0.79; *p* < 0.0001).

### Maternal serum CRP concentrations based on the presence of MIAC and/or IAI

**Fig 2:**
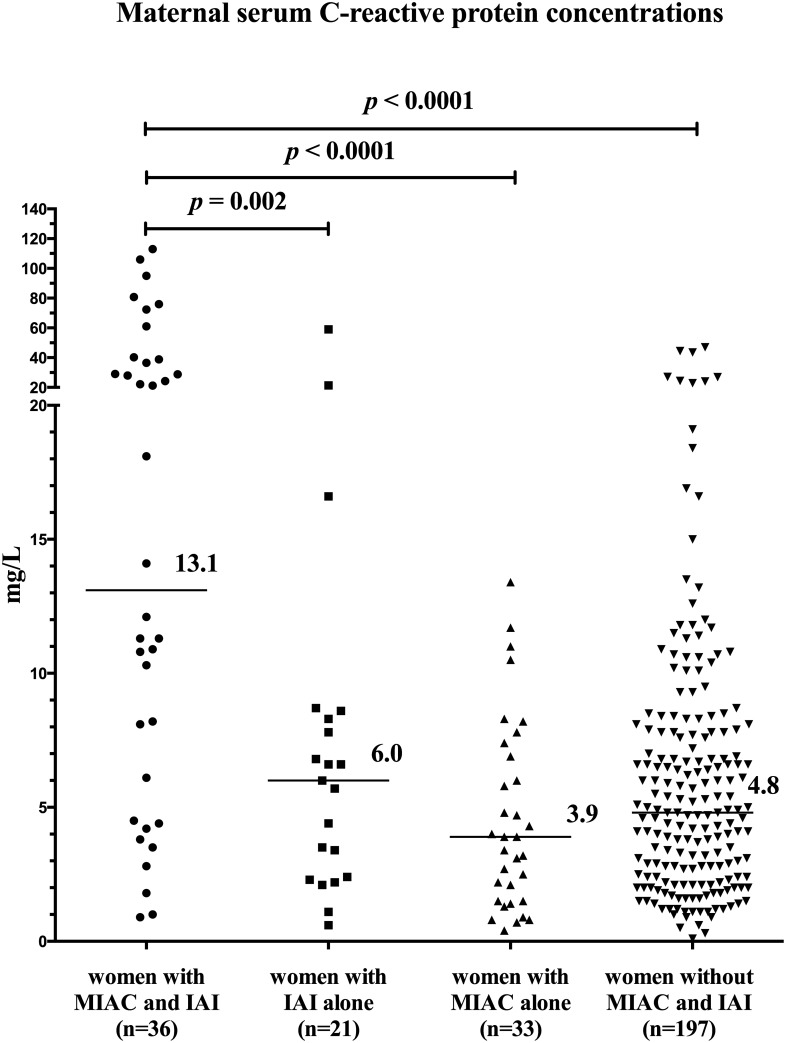
Maternal serum C-reactive protein concentrations in PPROM pregnancies complicated by the presence of MIAC and/or IAI. Women with both MIAC and IAI had higher maternal serum C-reactive protein concentrations than did women in other subgroups. Abbreviations: MIAC, microbial invasion of the amniotic cavity; IAI, intra-amniotic inflammation.

**Fig 3:**
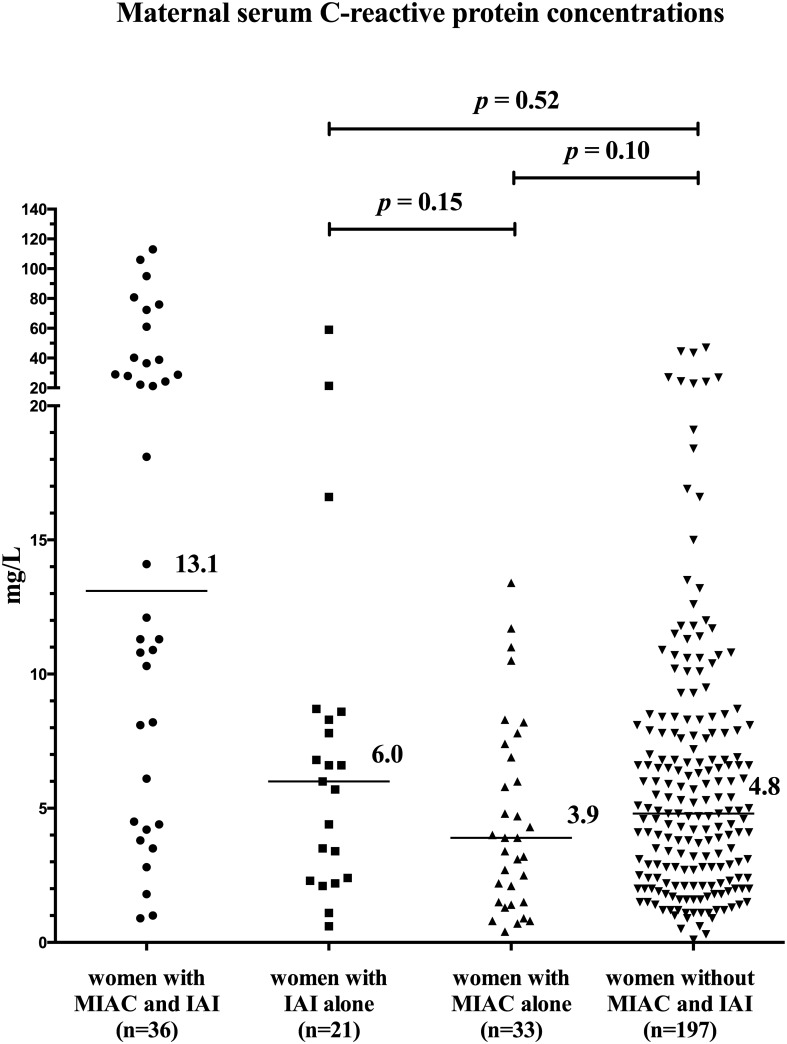
Maternal serum C-reactive protein concentrations in PPROM pregnancies complicated by the presence of MIAC and/or IAI. No differences were found among women with IAI alone, MIAC alone, and women without MIAC and IAI. Abbreviations: MIAC, microbial invasion of the amniotic cavity; IAI, intra-amniotic inflammation.

**Fig 4:**
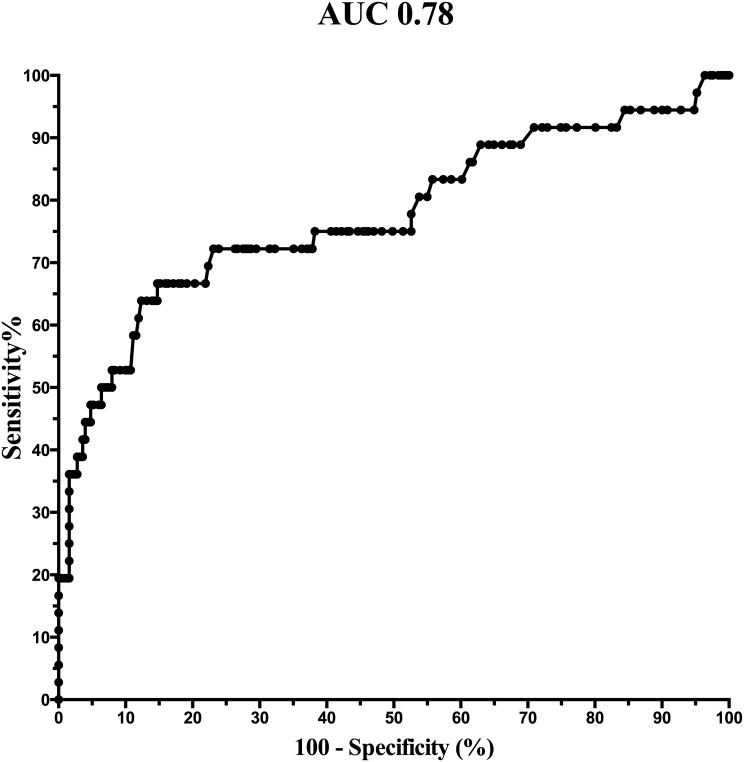
Maternal serum C-reactive protein concentrations with respect to the presence or absence of both MIAC and IAI. A receiver operating characteristic curve for the presence of both MIAC and IAI (area under the curve is 0.78 for IL-6 cutoff value > 17.5 mg/L; *p* < 0.0001). Abbreviations: MIAC, microbial invasion of the amniotic cavity; IAI, intra-amniotic inflammation.

Differences in maternal serum CRP concentrations were found among the four subgroups of women with PPROM based on the presence of MIAC and/or IAI (*p* < 0.0001) in crude analysis, as well as after adjustment for gestational age (*p* < 0.0001). Women with both MIAC and IAI had higher maternal serum CRP concentrations than women with IAI alone, MIAC alone, and those without MIAC and IAI in crude analysis (both MIAC and IAI: median: 13.1 mg/L, IQR 4.9–38.2; IAI alone: 6.0 mg/L, IQR 2.4–8.5; *p* = 0.002; MIAC alone: 3.9 mg/L, IQR 1.5–7.2; *p <* 0.0001; and without MIAC and IAI: median 4.8 mg/L, IQR 2.4–7.8; *p* < 0.0001; Fig 2), as well as after adjustment for gestational age at sampling, parity and BMI (IAI alone: *p* = 0.01; MIAC alone: *p =* 0.003; without MIAC and IAI: *p <* 0.0001). No differences in maternal serum CRP concentrations were detected among women with IAI alone, MIAC alone, and without MIAC and IAI (IAI alone vs. MIAC alone: *p* = 0.15; IAI alone vs. without MIAC and IAI: *p* = 0.52; MIAC alone vs. without MIAC and IAI: *p* = 0.10; Fig 3). The maternal serum CRP cutoff value of 17.5 mg/L was found to be the most effective at identifying both MIAC and IAI, with sensitivity of 47% [17/36; 95% confidence interval (CI) 32–63%] specificity of 96% (220/230; 95% CI 76–90%), positive predictive value of 42% (17/27; 95% CI 25–60%), negative predictive value of 96% (10/239; 95% CI 90–99%), positive likelihood ratio of 10.9 (95% CI 5.4–21.8), negative likelihood ratio of 0.6 (95% CI 0.4–0.8), odds ratio of 19.8 (95% CI 7.9–48.9), and area under the receiver operating characteristic curve of 0.78 (95% CI 0.68–0.87; *p* < 0.0001; Fig 4).

### Amniotic fluid IL-6 concentrations and maternal serum CRP concentrations

**Fig 5:**
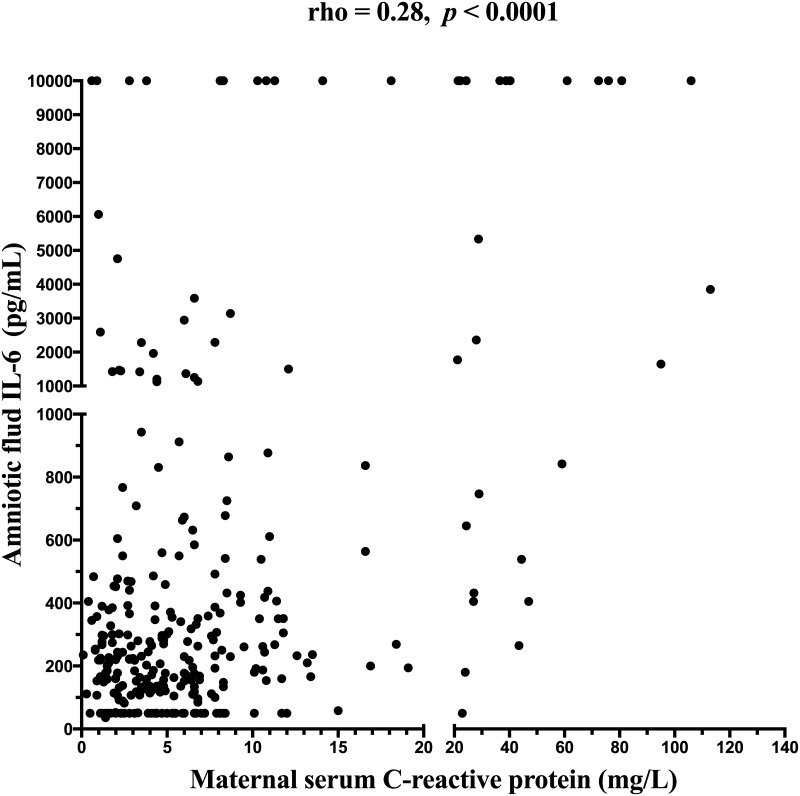
A correlation between maternal serum C-reactive protein and amniotic fluid IL-6 concentrations.

A weak positive correlation was identified between amniotic fluid IL-6 concentrations and maternal serum CRP concentrations (rho = 0.28, *p* < 0.0001; Fig 5).

## Discussion

Maternal serum CRP concentration is among the most commonly used clinical non-invasive markers to predict infectious-related and inflammatory complications in women with PPROM, in spite of the absence of strong evidence for its use in relation to these indications [16, 17]. However, there is a gap in knowledge, whether maternal serum CRP concentrations differs among women PPROM divided in the subgroups based on the presence and absence of MIAC and/or IAI.

In this study, we would like to bridge this gap in knowledge, and the following are the key findings of the present study: i) higher maternal serum CRP concentrations were associated with the presence of MIAC; ii) higher maternal serum CRP concentrations were associated with the presence of IAI; iii) the highest maternal serum CRP concentrations were found in women with both MIAC and IAI; iv) maternal serum CRP cutoff value of 17.5 mg/L was found to be most effective at identifying women with the presence of both MIAC and IAI; and v) maternal serum CRP concentrations weakly correlated with amniotic fluid IL-6 concentrations.

The pioneering papers regarding the associations between maternal serum CRP concentration and infectious-related and inflammatory complications, mainly clinical and histological chorioamnionitis, in PPROM have been published in nineties’ of the last century [27–29]. Nevertheless, the first paper to report on maternal serum CRP concentration and MIAC in PPROM was published by Romero’s group in 1996, which reported higher maternal serum CRP concentration in women with MIAC [30]. This finding was confirmed by several other studies [18, 31], however, the recent study by Cobo et al. did not show any differences in maternal serum CRP concentration between women with and without MIAC [32]. Our results from this relatively large cohort of women with PPROM supported the finding that the presence of MIAC is associated with higher maternal serum CRP concentrations. However, maternal serum CRP concentration is a poor predictor of the occurrence of MIAC.

Amniotic fluid IL-6 and matrix metalloproteinase 8 have been traditionally considered as the markers for the identification of IAI [7, 33–35]. Since obstetricians managing women with PPROM urgently require these results to assist in their decision-making process, point-of-care versions of these tests have been used in clinical practice [26, 36, 37]. Given that the cutoff value for IAI in point-of-care test for amniotic fluid IL-6 concentration was 745 pg/mL, we found that women with the presence of IAI had higher maternal serum CRP concentrations than women without IAI. These results were in keeping with the findings of Park et al., in spite of the fact that their definition of IAI was slightly different than ours; Park et al. defined the presence of MIAC and/or amniotic fluid IL-6 concentration as measured by ELISA ≥ 2600 pg/mL [31]. In our study, we have found that maternal serum CRP has limited diagnostic indices to identify the presence of IAI.

In our previous cohort of women with PPROM, we showed that women with both MIAC and HCA had the highest maternal serum CRP concentrations [18]. Likewise, when women from this cohort were split into four subgroups based on the presence and/or absence of MIAC and IAI, the subgroup with the presence of both MIAC and IAI had the highest maternal serum CRP concentrations. This indicated that the presence of both MIAC and IAI led to the highest maternal inflammatory response. This finding was especially interesting as IAI has been known to be associated with the highest fetal inflammatory response, independent of the presence or absence of MIAC. This means that the microbial origin of IAI is important for the inflammatory response in intra-amniotic and maternal, but not in fetal, compartments [5, 38].

Several studies have suggested maternal serum CRP concentration of 8 mg/L as a cutoff value for infection-related complications such chorioamnionitis, funisitis, and early-onset sepsis [39–41]. This cutoff has been considered valuable for its negative predictive value. In this study, we found that a maternal serum CRP cutoff value of 17.5 mg/L was ideal to identify the presence of both MIAC and IAI. This cutoff value had a very good specificity and negative predictive value. However, this cutoff value reached an AUC of 78%, which should be considered as just fair. In contrast, it is important to mention that our recently suggested cervical fluid IL-6 cutoff value of 500 mg/mL for the identification of both MIAC and IAI achieved the same AUC. Therefore, maternal serum CRP concentration should be still considered as a potentially non-invasive marker to identify, or mainly rule out, specific PPROM complications.

IL-6 is believed to be the primary trigger of CRP release [12, 14]. Based on this association, we decided to evaluate the correlation between amniotic fluid IL-6 and maternal serum CRP concentrations. Despite the fact that these markers were assessed in the different compartments a weak positive correlation was found.

A strong point of this study is the fact that only women with a clearly defined, specific phenotype of spontaneous preterm delivery (PPROM) were included. Second, maternal serum and amniotic fluid samples were obtained simultaneously at the time of admission. Therefore, the maternal CRP concentrations were compared with the conditions reflecting actual amniotic fluid status. Third, since maternal serum CRP concentrations have been shown to be influenced by the gestational age at sampling, parity, and BMI, the results were adjusted for these potential confounders. Another strength of this study was the relatively large cohort of women with PPROM. Several limitations of this study should be acknowledged. First, while this study focused on the association between a single measurement of maternal CRP concentrations at the time of admission and infection-related and inflammatory intra-amniotic complications, it did not take into consideration the trend of CRP concentrations during latency. Second, we used a non-cultivation-based technique to detect amniotic fluid microorganisms in this study. Previously, DiGuilio et al. reported that in some cases with PPROM, some bacteria may be revealed by cultivation, even in the absence of 16S rRNA[3]. Therefore, we cannot rule out that the microbial-associated IAI and colonization groups might be underestimated, while the groups with sterile IAI and without both MIAC and IAI might be overestimated. Third, we did not evaluate amniotic fluid CRP concentrations to determine whether there was an association between maternal and amniotic fluid CRP concentrations.

In conclusion, maternal serum CRP at the time of admission can rule out the presence of both MIAC and IAI, therefore, it may serve as a non-invasive screening tool to distinguish between women with PPROM who are at high or at low risk for the presence of both MIAC and IAI.

## Supporting information

S1 Dataset (XLSX)
